# *Staphylococcus aureus* Isolated From Retail Meat and Meat Products in China: Incidence, Antibiotic Resistance and Genetic Diversity

**DOI:** 10.3389/fmicb.2018.02767

**Published:** 2018-11-15

**Authors:** Shi Wu, Jiahui Huang, Qingping Wu, Jumei Zhang, Feng Zhang, Xiaojuan Yang, Haoming Wu, Haiyan Zeng, Moutong Chen, Yu Ding, Juan Wang, Tao Lei, Shuhong Zhang, Liang Xue

**Affiliations:** ^1^Guangdong Institute of Microbiology, State Key Laboratory of Applied Microbiology Southern China, Guangdong Provincial Key Laboratory of Microbial Culture Collection and Application, Guangdong Open Laboratory of Applied Microbiology, Guangzhou, China; ^2^School of Bioscience and Bioengineering, South China University of Technology, Guangzhou, China; ^3^Department of Food Science & Technology, Jinan University, Guangzhou, China; ^4^College of Food Science, South China Agricultural University, Guangzhou, China

**Keywords:** *S. aureus*, retail meat, antimicrobial resistance, MLST, *spa* typing

## Abstract

This study was to estimate the prevalence and characteristics of *Staphylococcus aureus* from 1,850 retail meat and meat products in China during July 2011 to June 2016. The samples were collected covering most provincial capitals in China, including 604 raw meat, 601 quick-frozen meat, and 645 ready-to-eat meat. Using the qualitative and quantitative methods, all 39 cities had *S. aureus*-positive samples, and *S. aureus* was detected in 35.0% (647/1,850) of the samples. The levels of *S. aureus* in retail meat showed that the MPN value of the majority of the positive samples ranged from 0.3 to 100 MPN/g. Twenty-four antibiotics were used to test all 868 *S. aureus* isolates for antibiotic susceptibility. Only 11 isolates (1.26%) were susceptible to all antibiotics, whereas most isolates (821/868, 94.6%) showed resistance or intermediary resistance to more than three or more antibiotics. Of these strains, 104 (12.0%) were resistant to more than 10 antibiotics. However, the most frequent resistance was observed to ampicillin (85.4%), followed by penicillin (84.6%), erythromycin (52.7%), tetracycline (49.3%), kanamycin (45.3%), telithromycin (30.1%), clindamycin (29.6%), streptomycin (21.1%), norfloxacin (20.4%), gentamicin (19.4%), fusidic acid (18.4%), ciprofloxacin (16.9%), chloramphenicol (13.1%), amoxycillin/clavulanic acid (11.0%), and others (<10%). 7.4% of isolates (62/868) were confirmed as methicillin-resistance *S. aureus* (MRSA). By molecular typing analysis, there were 164 *spa* types and 111 STs were identified, including 15 novel *spa* types and 65 newly STs by multilocus sequence typing (MLST) and *spa* typing. Despite the wide genetic diversity observed among the 868 isolates, a great proportion of the population belonged to finite number of major clones: ST1-t127 (93/868, 10.7%) and ST7-t091 (92/868, 10.6%), ST5-t002 (42/868, 4.8%), ST398-t034 (40/868, 4.6%), ST188-t034 (38/868, 4.4%), ST59-t437 (30/868, 3.5%), ST6-t701 (29/868, 3.3%), and ST9-t899 (27/868, 3.1%) in China. This study reflects *S. aureus* was readily detected in Chinese retail meat and meat products but the level were not very excessive. In this study, the high antibiotic resistance is alarming and raising public health concern. In additions, most of molecular types of isolates have been linked to human infections around the world, indicating that these types of *S. aureus* in China have a theoretical pathogenic potential.

## Introduction

Recent years, many pathogens were responsible for food safety. *Staphylococcus aureus* is recognized as one of the major foodborne pathogens in fresh and ready-to-eat products and responsible for various infections around world (Diep et al., 2006). It could grows at temperature between 15°C and 45°C and at NaCl concentrations as high as 15% (Behling et al., 2010). This bacterium multiplies quickly at room temperature to produce toxins that cause illness. Naturally, the distribution of *S. aureus* was ubiquity in the world, but the most important infection source of *S. aureus* was food. Every year, *S. aureus* involved in about 241,000 illnesses of foodborne disease in USA (Scallan et al., 2011; Wu et al., 2018). In 2013, there were 12.5% of foodborne bacterial outbreaks are caused by *S. aureus* in China, which showed third most frequently pathogen after *Vibrio parahaemolyticus* (27.8%) and *Salmonella* (23.1%) (Wei-Wei et al., 2018).

In the last decades, the spread use of antibiotics in bacteria is increasing the emergence of multidrug resistant strains (MDR), which showed great challenges to public health. As a formidable adaptive capacity strain, *S. aureus* can adapt to varying environmental conditions and rapidly become resistant to virtually all antibiotics (Mccallum et al., 2010). Nowadays, more and more MDR *S. aureus* were reported in food poisoning outbreaks and isolated from food product in previous researched (Sauer et al., 2008; Huang et al., 2009; Gharsa et al., 2012; Papadopoulos et al., 2018). Moreover, in recent years, methicillin-resistance *S. aureus* (MRSA) is attracting extensive attention. It usually showed multiple antimicrobial resistance and listed as one of 12 families of bacteria that pose the greatest threat to human health by WHO in 2017 (Govindaraj and Vanitha, 2018).

Nowadays, various of molecular subtyping approaches [e.g., pulsed-field gel electrophoresis (PFGE), multilocus sequence typing (MLST), and staphylococcal protein A (*spa*) typing] have been developed for the characterization of *S. aureus*. For epidemiology and evolutionary studies, as well as discriminatory power, it is important to produce unambiguous results that are readily comparable among different laboratories, and it is necessary to have a system for classifying the relationships among closely related strains to monitor changes and patterns in clonal lineages over time or space (O'Hara et al., 2016). Of these methods, MLST and *spa* typing showed highly clonal population structure identified for *S. aureus* in many previous study (Kanika et al., 2011; Fetsch et al., 2014; Basanisi et al., 2017). As a widely accepted method, MLST, which is based on DNA sequence and relied on analysis of relatively conserved genes that encode essential proteins, has proven very useful for epidemiology and evolutionary studies (Saunders and Holmes, 2007). *spa* typing is another efficient typing method for *S. aureus*, it based on sequencing of the polymorphic X region of the protein A gene (*spa*) (Hallin et al., 2009). It showed highly concordant with MLST. Some studies suggest that it is suitable for epidemiology and evolutionary investigations based on studies of European and international isolates (Koreen et al., 2004; Hallin et al., 2007; Strommenger et al., 2008). However, other studies question the use of a single locus method such as spa typing for epidemiologic investigations since recombination events might distort the underlying clonal relationships (O'Hara et al., 2016). Both two methods can assigned to MLST STs and *spa* types, which easily to compare with other laboratories in the world.

Of various food products surveyed, meat and meat products are widely known to be an important reservoir for *S. aureus* and involved in several outbreaks (Aydin et al., 2011; Hennekinne et al., 2012; Wang et al., 2013; Sallam et al., 2015). The investigate of *S. aureus* from meat can implement a system monitoring. In China, the qualitative and quantitative data of this bacterium in retail meat from different areas were limit. Therefore, the prevalence and levels of *S. aureus* from South China to North China was to investigate in retail meat and meat products in this study. After isolation and identification, antibiotic susceptibility test, as well as *spa* typing and MLST were used to determine the genetic background among the *S. aureus* isolates.

## Materials and methods

### Sample collection

**Figure 1:**
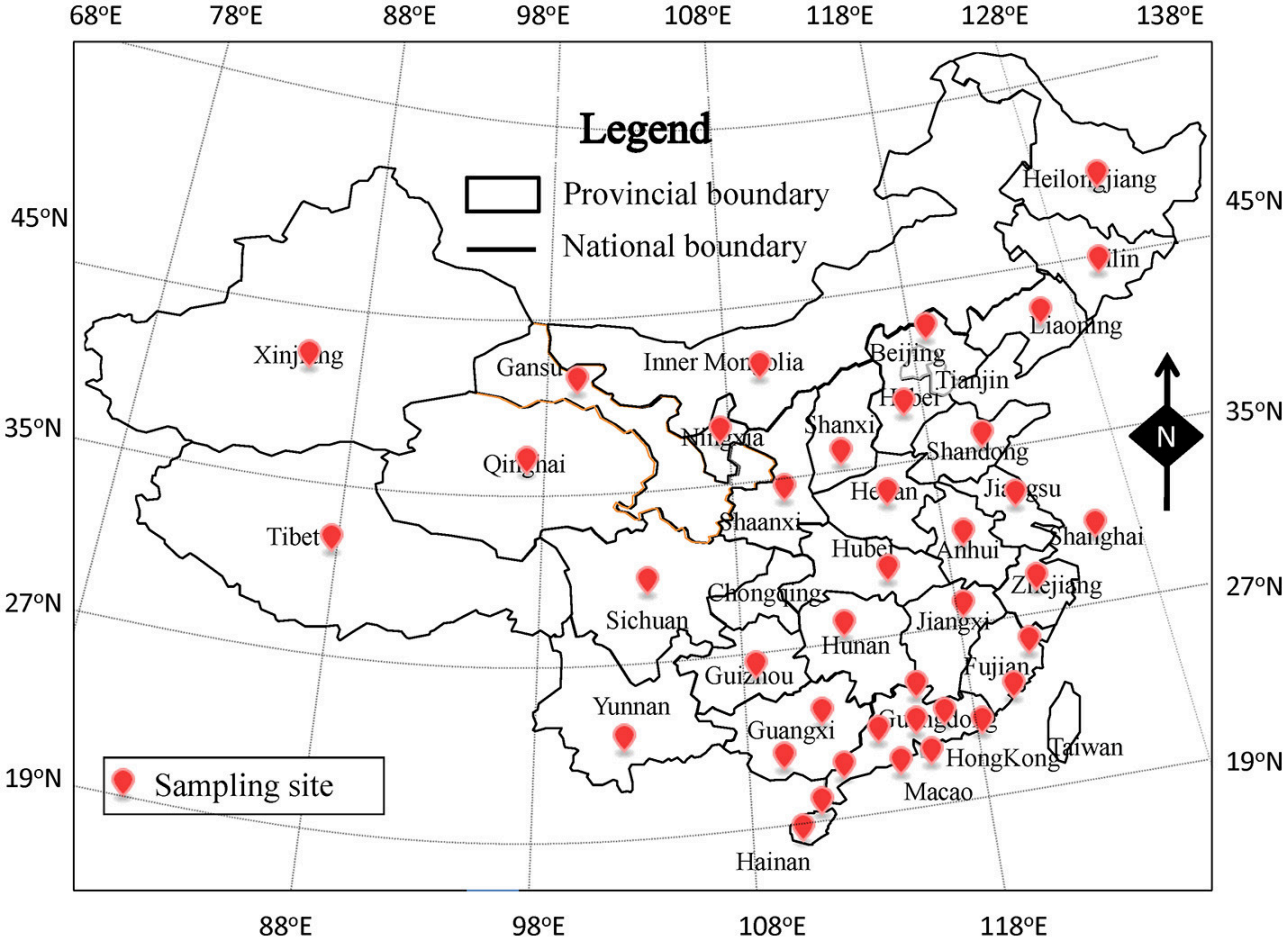
The location of sampling sites in China of this study.

From July 2011 to June 2016, a total of 1,850 retail meat samples were collected from supermarkets, fairs, and farmers' markets. The samples, including 604 raw meat (bacon/sausage, poultry, pork, mutton, and beef), 601 quick-frozen meat (frozen dumpling/steamed stuffed bun, frozen poultry, frozen pork, frozen mutton, and frozen beef), and 645 ready-to-eat (RTE) meat (roast chicken/duck, salt-baked chicken, stewed meat sausage, and ham), were obtained from 39 cities, which covered most of provincial capitals of China (Figure 1). All collected samples were tightly sealed with sterile plastic wrap and placed in a cold box at a temperature lower than 4°C, then transported to an accredited laboratory and subjected to microbiological analysis within 24 h.

### Isolation and identification of *S. aureus*

The examination of *S. aureus* proceeded according to GB 4789.10-2010 of food microbiological (National Food Safety Standards of China) with slight modification. Briefly, 25 g of food sample was homogenized in 225 mL of tryptic soy broth with 10% sodium chloride (Huankai, Guangzhou, China). Subsequently, 1, 0.1, and 0.01-mL aliquots were transferred to tubes containing 9, 10, and 10 mL in triplicate with trypticase soy broth (Huankai) supplemented with 10% NaCl. Each tubes were incubated at 37°C for 48 h, respectively. A loopful of enrichment broth culture (10 μL) was streaked onto chromogenic *S. aureus* agar plates (Huankai) and incubated at 37°C for 24 h. One to four colonies with pink color were purified on nutrient agar medium. The purified colonies were analyzed via coagulase activity test by freeze-dried Rabbit Plasma (Huankai), and the API STAPH test strips (bio Merieux, Marcy-1'Etoile, France) was used. The MPN value was determined on the basis of the number of positive tube(s) in each of the three sets using the MPN table.

### Antibiotic susceptibility testing

All *S. aureus* isolates were tests for antibiotic susceptibility by the disk diffusion method using Mueller–Hinton agar (Bauer et al., 1966) and commercially available discs (Oxoid, UK). The antimicrobial agents used were amoxycillin/clavulanic acid, ampicillin, cefepime, cefoxitin, penicillin G, ceftazidime, amikacin, gentamicin, kanamycin, streptomycin, chloramphenicol, clindamycin, erythromycin, telithromycin, ciprofloxacin, norfloxacin, tetracycline, linezolid, trimethoprim/sulphamethoxazole 1:19, rifampicin, quinupristin/dalfopristin, teicoplanin, nitrofurantoin, and fusidic acid. MICs for linezolid resistant isolates by disk diffusion were also confirmed by the agar dilution method on Mueller–Hinton agar. The results were scored according to the guidelines of the Clinical and Laboratory Standards Institute (CLSI, 2015). *Staphylococcus aureus* ATCC25923 and *Escherichia coli* ATCC25922 were included as a control. The presence of the *mecA/mecC* gene was studied by PCR in all cefoxitin-resistant isolates (Perez-Roth et al., 2001; Stegger et al., 2012).

### *spa*- and multi locus sequence typing

All isolates were analyzed by *spa* typing and MLST for genetic diversity analysis. Primers spa-1113f (5′-TAA AGA CGA TCC TTC GGT GAG C-3′) and spa-1514r (5′-CAG CAG TAG TGC CGT TTG CTT-3′) were used for *spa* amplification in each strains (Shopsin et al., 1999). The MLST scheme was followed using seven housekeeping genes: *arcC, aroE, glpF, gmk, pta, tpi*, and *yqil*. PCR products generated by both techniques were purified with a PCR purification kit (Qiagen, Genman) and were sequenced at an ABI 3730XL sequencer (Applied BioSystems). *Spa* types were assigned with the SpaServer website (http://spaserver2.ridom.de). The protocols was conducted using previous described primers (Enright et al., 2013). Sequence types (STs) were identified by consulting the *S. aureus* MLST database (https://pubmlst.org/saureus/). The clonal complex (CC) analysis was performed using the eBURST algorithm as previously described (Feil et al., 2004). The minimum spanning tree (MST) was constructed with Bionumerics 7.6 software (Applied Maths, Sint-Martens-Latem, Belgium).

### Statistical analysis

For statistical analysis, the bacterial numbers were converted to base-10 logarithms. MPN values < 0.3 MPN/g were set to 0.15, and MPN values > 110 MPN/g were assigned the maximum value (Motes et al., 1998). The data of prevalence and levels of *S. aureus-*positive samples sorted by different food type and sampling sites were compared by using the analysis of variance (SPSS v21.0). Differences between the mean values were significant when *P* < 0.05.

## Results

### Prevalence and levels of *S. aureus* in retail meat in china

In this survey, a total of 1,850 samples were collected in 39 Chinese cities. Overall, there were 647 samples showed coagulase positive and confirmed for *S. aureus* by API STAPH test. The average incidence of *S. aureus* was 35.0% (647/1,850), the MPN value of the majority of the positive samples ranged from 0.3 to 110 MPN/g, which showed the geometric mean was 10.35 MPN/g. Out of the 647 positive *S. aureus* samples, 33 (5.1%) exceeded 100 MPN/g, whereas 319 (49.3%) were below 1 MPN/g.

The analyzed food products were classified into three categories (raw meat, quick-frozen meat, and ready-to-eat meat), and the values of *S. aureus* contamination in each sample were determined (Table 1). Among the analyzed categories, raw meat was the most frequently contaminated with *S. aureus*, with a prevalence that reached 51.0%. The prevalence of *S. aureus* in raw poultry, raw mutton, raw beef, raw pork and bacon/sausage was 67.9, 54.5, 50.4, and 18.6%, respectively. The quick-frozen meat showed 43.4% of contamination for *S. aureus* in this survey, including 60.9% of positive samples in quick-frozen poultry, 50.0% in quick-frozen pork, 31.4% in quick-frozen beef, 30.9% in quick-frozen mutton, and 29.4% in quick-frozen dumpling. The prevalence of *S. aureus* in RTE meat was lower than that in other samples, which detected 12.2% of RTE samples were positive for *S. aureus*. *S. aureus* were detected in 25.0% (3/25) of RTE beef, 12.7% (22/173) of RTE pork, and 11.8% (54/456) of RTE poultry, whereas RTE mutton was free of *S. aureus*. Combining the MPN values, raw meat showed the highest levels of *S. aureus* (14.05 MPN/g), followed by RTE meat (9.11 MPN/g) and quick-frozen meat (6.37 MPN/g). Most of the *S. aureus*-positive samples exhibited less than 10 MPN/g, such as RTE poultry (9.89 MPN/g), quick-frozen poultry (7.37 MPN/g), pork (6.67 MPN/g), RTE pork (6.24 MPN/g), quick-frozen dumpling (4.99 MPN/g), quick-frozen mutton (1.96 MPN/g), quick-frozen pork (1.14 MPN/g), and bacon/sausage (0.59 MPN/g).

**Table 1:** Occurrence and levels of *Staphylococcus aureus* in different types of retail meat.

**Types of product**	**No. of samples**	**No. (%) of positive samples**		**No. of positive samples by quantitative methods by MPN/g range**				***S. aureus* level^b^ (MPN/g)**
				**MPN < 1**	**1 ≤ MPN < 10**	**10 ≤ MPN < 100**	**MPN ≥100**	
Raw meat	604	308	51.0%	125	121	37	25	14.05
Pork	239	114	47.7%	54	45	13	2	6.76
Beef	125	63	50.4%	31	22	6	4	10.27
Poultry	159	108	67.9%	25	48	18	17	23.48
Mutton	22	12	54.5%	5	5	0	2	20.18
Bacon/sausage	59	11	18.6%	10	1	0	0	0.59
Quick-frozen meat^a^	601	260	43.3%	150	81	24	5	6.37
Quick-frozen pork	10	5	50.0%	4	1	0	0	1.14
Quick-frozen beef	35	11	31.4%	5	2	4	0	11.84
Quick-frozen poultry	253	154	60.9%	82	53	15	4	7.37
Quick-frozen mutton	68	21	30.9%	16	4	1	0	1.96
Quick-frozen dumpling	235	69	29.4%	43	21	4	1	4.99
RTE meat	645	79	12.2%	44	24	8	3	9.11
RTE pork	173	22	12.7%	14	7	0	1	6.24
RTE beef	12	3	25.0%	1	1	1	0	15.95
RTE poultry	456	54	11.8%	29	16	7	2	9.89
RTE mutton	4	0	0.0%	0	0	0	0	0.00
Total	1850	647	35.0%	319	226	69	33	10.35

a *All quick-frozen meat were stored at −10°C before being sold*.

b *The values are the weighted averages, shown as the geometric means of the positive samples*.

The distribution of *S. aureus* among different sampling sites is shown in Table 2. However, all of the 39 cities had *S. aureus*-positive samples, ranging from 16.7% in Shenzhen to 48.8% in Beihai. The prevalence of *S. aureus* exceeded 40% in one third of sampling sites. The highest contamination level in Beihai was 20.94 MPN/g, which showed 47.6% (10/21) of the positive samples were exceeded 10 MPN/g, whereas the lowest level in Changsha was only 0.43 MPN/g, which showed 80.0% (8/10) of the positive samples were less than 1 MPN/g. A total of 1,201 samples were collected from South China, with 35.3% prevalence, and 26 positive samples (26/424, 6.1%) had *S. aureus* densities exceeding the detectable level of 100 MPN/g. In North China, *S. aureus* was detected in 223 out of 649 samples (34.4%), of which only 7 samples (3.1%) of these samples had levels exceeding 100 MPN/g, whereas 127 samples (57.0%) yielded <1 MPN/g. Thus, there is no significantly difference (*p* > 0.05, χ^2^ test) between South China and North China.

**Table 2:** Prevalence and levels of *Staphylococcus aureus* at different sampling sites.

**Sampling site**		**No. of samples**	**No. (%) of positive samples**	**No. of positive samples by quantitative methods (MPN/g)**				***S. aureus* level^b^ (MPN/g)**
**City**	**Province**			**MPN < 1**	**1 ≤ MPN < 10**	**10 ≤ MPN < 100**	**MPN ≥100**	
Guangzhou	Guangdong	212	66 (31.1)	22	26	13	5	14.46
Shenzhen	Guangdong	42	7 (16.7)	2	3	1	1	19.4
Shaoguan	Guangdong	42	9 (21.4)	4	5	0	0	1.71
Zhanjiang	Guangdong	42	18 (42.9)	10	5	3	0	6.62
Shantou	Guangdong	42	11 (26.2)	0	9	1	1	15.65
Heyuan	Guangdong	45	14 (31.1)	8	3	2	1	11.8
Haikou	Hainan	43	16 (37.2)	6	7	1	2	19.07
Sanya	Hainan	43	20 (46.5)	12	6	2	0	3.34
Beihai	Guangxi	43	21 (48.8)	5	6	8	2	20.94
Nanning	Guangxi	42	20 (47.6)	6	10	4	0	7.83
Fuzhou	Fujian	41	20 (48.8)	6	8	4	2	18.58
Xiamen	Fujian	43	18 (41.9)	6	9	2	1	11.13
Macao^a^	–	47	16 (34.0)	7	5	3	1	13.85
Hongkong^a^	–	46	19 (41.3)	11	5	2	1	10.23
Shanghai^a^	–	44	16 (36.4)	8	7	1	0	5.01
Hefei	Anhui	43	11 (25.6)	7	2	2	0	8.39
Nanchang	Jiangxi	41	19 (46.3)	9	7	2	1	11.63
Wuhan	Hubei	41	15 (36.6)	6	5	2	2	19.5
Chengdu	Sichuan	43	20 (46.5)	8	8	2	2	16.91
Kunming	Yunnan	42	14 (33.3)	6	6	1	1	12.71
Changsha	Hunan	44	10 (22.7)	8	2	0	0	0.43
Hangzhou	Zhengjiang	44	15 (34.1)	12	1	1	1	11.78
Guiyang	Guizhou	43	14 (32.6)	12	1	0	1	8.43
Nanjing	Jiangsu	43	15 (34.9)	11	2	1	1	11.04
South China		1201	424 (35.3)	192	148	58	26	12.23
Lanzhou	Gansu	44	14 (31.8)	7	7	0	0	2.18
Haerbin	Heilongjiang	42	18 (42.9)	12	6	0	0	1.31
Xi'an	Shaanxi	42	15 (35.7)	6	6	1	2	18.35
Taiyuan	Shanxi	41	14 (34.2)	7	6	1	0	2.58
Beijing^a^	–	41	11 (26.8)	6	5	0	0	1.37
Jinan	Shandong	41	18 (43.9)	8	9	0	1	7.81
Changchun	Jilin	45	17 (37.8)	10	5	2	0	4.99
Xining	Qinghai	44	15 (34.1)	14	0	1	0	3.28
Yinchuan	Ningxia	45	10 (22.2)	7	2	0	1	11.52
Huhehaote	Neimenggu	44	15 (34.1)	9	5	1	0	2.36
Shenyang	Liaoning	45	19 (42.2)	9	8	0	1	8.39
Shijiazhuang	Hebei	44	16 (36.4)	10	5	1	0	3.73
Zhengzhou	Henan	43	10 (23.3)	6	3	1	0	3.8
Lasa	Tibet	44	20 (45.5)	10	6	3	1	10.07
Wulumuqi	Xinjiang	44	11 (25.0)	6	4	0	1	11.78
North China		649	223 (34.4)	127	77	11	7	6.25

a *These cities are direct-controlled municipalities*.

b *The values are weighted averages shown as the geometric means of positive samples*.

### Antibiotic susceptibility and detection of *mecA* of isolates

A total of 868 isolates (1–2 isolates per sample) were collected from the retail meat and meat products, consisting of 415 isolates from raw meat, 354 isolates from quick-frozen meat, and 99 isolates from RTE meat. The results of antibiotic susceptibility testing of all isolates are shown in Table 3. Overall, only 11 isolates (1.26%) were susceptible to all 24 tested antibiotics, whereas most isolates (821/868, 94.6%) showed resistance or intermediary resistance to more than three or more antibiotics. Of these isolates, 104 isolates (12.0%) were resistant to more than 10 antibiotics. For 24 antibiotics, 85.4% of isolates were resistant to ampicillin, followed by penicillin G (84.6%), erythromycin (52.7%), tetracycline (49.3%), kanamycin (45.3%), telithromycin (30.1%), clindamycin (29.6%), streptomycin (21.1%), norfloxacin (20.4%), gentamicin (19.4%), fusidic acid (18.4%), ciprofloxacin (16.9%), chloramphenicol (13.1%), amoxycillin/clavulanic acid (11.0%), and others (<10%). There is no significant difference (*p* > 0.05, χ^2^ test) between the resistances of types of meat samples for most antimicrobials tested. In total, 95.5, 92.1, and 94.0% of the *S. aureus* isolates from raw meat, quick-frozen meat, and RTE meat were resistant to at least one antimicrobial. In this study, it was found 62 MRSA isolates (7.14%) which showed resistant to cefoxitin and carried *mecA* genes, including 34 isolates (8.2%) from raw meat, 20 isolates (5.6%) from quick-frozen meat, and 8 isolates (8.1%) from RTE meat. The MRSA isolates were resistant to most selected β-lactams and other antibiotics, and 77.4% (48/62) of them were resistant to more than 10 antibiotics.

**Table 3:** Antimicrobial resistance of *Staphylococcus aureus* isolated from retail meat and meat product in China.

**Antimicrobial group**	**Antibiotics**	**No. (%) of resistant isolates**			
		**All isolates (*n* = 868)**	**Raw meat (*n* = 415)**	**Quick-frozen meat (*n* = 354)**	**RTE meat (*n* = 99)**
β-Lactams	Amoxicillin/clavulanic acid	96 (11.1)	46 (11.1)	39 (11.0)	11 (11.1)
	Ampicillin	742 (85.4)	361 (87.0)	293 (82.8)	88 (88.9)
	Cefepime	46 (5.3)	23 (5.5)	17 (4.8)	6 (6.1)
	Cefoxitin	62 (7.1)	34 (8.2)	20 (5.6)	8 (8.1)
	Penicillin G	735 (84.6)	357 (86.0)	289 (81.6)	89 (89.9)
	Ceftazidime	68 (7.8)	37 (8.9)	23 (6.5)	8 (8.1)
Aminoglycosides	Amikacin	65 (7.5)	33 (8.0)	28 (7.9)	4 (4.0)
	Gentamicin	169 (19.4)	76 (18.3)	67 (18.9)	26 (26.3)
	Kanamycin	394 (45.3)	207 (49.9)	144 (40.7)	43 (43.4)
	Streptomycin	183 (21.1)	83 (20.0)	81 (22.9)	19 (19.2)
Phenicols	Chloramphenicol	114 (13.1)	64 (15.4)	41 (11.6)	9 (9.1)
Lincosamides	Clindamycin	257 (29.6)	122 (29.4)	111 (31.4)	24 (24.2)
Macrolides	Erythromycin	458 (52.7)	222 (53.5)	190 (53.7)	47 (47.5)
	Telithromycin	262 (30.1)	124 (29.9)	108 (30.5)	30 (30.3)
Fluoroquinolones	Ciprofloxacin	147 (16.9)	77 (18.6)	61 (17.2)	8 (8.1)
	Norfloxacin	177 (20.4)	87 (21.0)	81 (22.9)	8 (8.1)
Tetracyclines	Tetracycline	428 (49.3)	227 (54.7)	169 (47.7)	32 (32.3)
Oxazolidinones	Linezolid	5 (0.6)	0 (0.0)	1 (0.1)	4 (0.5)
Ansamycins	Rifampicin	81 (9.3)	34 (8.2)	32 (9.0)	15 (15.2)
Sulfonamides	Trimethoprim/sulphamethoxazole 1:19	51 (5.9)	27 (6.5)	20 (5.6)	4 (4.0)
Quinolones	Quinupristin/dalfopristin	34 (3.9)	15 (3.6)	15 (4.2)	4 (4.0)
Glycopeptides	Teicoplanin	8 (0.9)	5 (1.2)	3 (0.8)	0 (0.0)
Nitrofurantoins	Nitrofurantoin	10 (1.2)	8 (1.9)	2 (0.6)	0 (0.0)
	Fusidic acid	160 (18.4)	73 (17.6)	60 (16.9)	26 (26.3)
**ANTIMICROBIALS**					
	1–5 Antimicrobials	478 (55.0)	234 (56.4)	192 (54.2)	55 (55.6)
	6–10 Antimicrobials	234 (26.9)	114 (27.5)	93 (26.3)	30 (30.3)
	11–15 Antimicrobials	79 (9.1)	34 (8.2)	33 (9.3)	7 (7.1)
	16–24 Antimicrobials	25 (2.9)	14 (3.4)	8 (2.3)	1 (1.0)

### Molecular typing of *S. aureus*

By the *spa* typing methods, we identified 164 different *spa* types including 15 novel *spa* types that were not in the Ridom StaphType database among the 868 isolates tested. Four isolates could not be typed (Table 4). The most commonly observed *spa* types were t091 (113/868, 13.0%) and t127 (104/868, 12%), followed by t002 (49/868, 5.7%), t189 (47/868, 5.4%), t034 (46/868, 5.3%), t701 (33/868, 3.8%), t437 (30/868, 3.5%), t899 (29/868, 3.3%), t796 (27/868, 3.1%), t084 (19/868, 2.2%), t3092 (15/868, 1.7%), t085 (14/868, 1.6%), t164 (14/868, 1.6%), t1376 (13/868, 1.5%), t213 (9/868, 1.0%), and other *spa* types (<1.0%).

**Table 4:** The CCs, STs, and *spa* types of the 868 *S. aureus* isolated from retail meat and meat products.

**Clonal complex**	**STs (No.)**	***spa* types (No.)**
CC1 (133)	ST1 (124)	t127 (93), t4792 (7), t174 (2), t177 (2), t2459 (2), t286 (2), t3471 (2), t5500 (2), t2478 (1), t078 (1), t8619 (1), t286 (1), t13819 (1), t693 (1), t591 (1), t899 (1), t17635^*^ (1), t1908 (1), t9632 (1), t701 (1)
	ST573 (1)	t345 (1)
	ST1920 (1)	t286 (1)
	ST4446^*^ (2)	t127 (2)
	ST4477^*^ (1)	t1491 (1)
	ST4473^*^ (1)	t127 (1)
	ST4455^*^ (1)	t127 (1)
	ST4449^*^ (1)	t127 (1)
	ST4470^*^ (1)	t127 (1)
CC7 (174)	ST7 (151)	t091 (92), t796 (26), t1943 (3), t085 (3), t2616 (4), t289 (3)t10370 (3), t7568 (4), t10332 (1), t3932 (2), t605 (2), t3437 (2), t1689 (1), t304 (1), t3092 (1), t8927 (1), t1685 (1), t127 (1)
	ST943 (14)	t091 (11), t002 (1), t250 (1), t289 (1)
	ST4440^*^ (1)	t091 (1)
	ST4466^*^ (1)	t796 (1)
	ST4457^*^ (1)	t796 (1)
	ST4476^*^	t091 (2)
	ST4450^*^	t1685 (2)
	ST4442^*^ (1)	t091 (1)
	ST4465^*^ (1)	t091 (1)
CC15 (47)	ST15 (35)	t085 (8), t084 (12), t346 (3), t803 (4), t2036 (4), t360 (1), t2422 (1), t774 (1), t17638^*^ (1)
	ST199 (1)	t084 (1)
	ST3685 (1)	t346 (1)
	ST333 (1)	t084 (1)
	ST3055	t084 (4), t2055 (1)
	ST906 (1)	t085 (1)
	ST2445 (1)	t346 (1)
	ST4438^*^ (1)	t085 (1)
	ST4568^*^ (1)	t084 (1)
CC5 (85)	ST5 (73)	t002 (42), t3478 (5), t954 (5), t548 (4), t688 (2), t777 (2), t1265 (2), t601 (1), t8565 (1), t1107 (1), t071 (1), t034 (1), t2051 (1), t105 (1), t502 (1), No-type(3)
	ST950 (4)	t895 (4)
	ST2690 (1)	t777 (1)
	ST1635 (2)	t002 (2)
	ST965 (2)	t062 (2)
	ST403 (1)	t002 (1)
	ST4572^*^ (1)	t12870 (1)
	ST4070^*^ (1)	t002 (1)
CC188 (55)	ST188 (48)	t189 (38), t5229 (3), t10939 (2), t3887 (2),t2883 (1), t5229 (1), t1858 (1)
	ST1218 (1)	t189 (1)
	ST4570^*^ (1)	t189 (1)
	ST4103^*^ (1)	t189 (1)
	ST4441^*^ (1)	t189 (1)
	ST4072^*^ (1)	t189 (1)
	ST4064^*^ (1)	t189 (1)
	ST4458^*^ (1)	t189 (1)
CC398 (66)	ST398 (59)	t034 (40), t571 (5), t1451 (3), t1250 (2), t002 (2), t899 (1), t1456 (1), t011 (1), t1250 (1), t1255 (1), t9472 (1), t1928 (1)
	ST2392 (1)	t1255 (1)
	ST4439^*^ (1)	t12250 (1)
	ST4454^*^	t034 (2)
	ST4567^*^ (1)	t034 (1)
	ST4074^*^ (1)	t034 (1)
	ST4068^*^ (1)	t034 (1)
CC6 (47)	ST6 (42)	t701 (29), t091 (2), t4562 (1), t304 (4), t3802 (2), t11164 (1), t605 (1), t4793 (1), t034 (1)
	ST2114 (1)	t701 (1)
	ST4460^*^ (1)	t934 (1)
	ST4061^*^ (1)	t701 (1)
	ST4453^*^ (1)	t701 (1)
	ST4571^*^ (1)	t085 (1)
CC4628 (4)	ST4628^*^ (1)	t11641 (1)
	ST4627^*^ (1)	t11641 (1)
	ST4626^*^ (1)	t11641 (1)
	ST4077^*^ (1)	t11641 (1)
CC20 (15)	ST1281 (9)	t164 (9)
	ST20 (5)	t164 (4), t1987 (1)
	ST2631 (1)	t164 (1)
CC59 (44)	ST59 (42)	t437 (30), t1751 (4), t441 (2), t163 (3), t127 (1), t3485 (1), t543 (1)
	ST338 (1)	t4911 (1)
	ST3355 (1)	t3485 (1)
CC30 (7)	ST30 (3)	t021 (1), t964 (1), t018 (1)
	ST4462^*^ (3)	t1397 (3)
	ST4478^*^ (1)	t11012 (1)
CC522 (8)	ST522 (6)	t14061 (1), t5428 (3), t7630 (2)
	ST4474^*^ (1)	t14061 (1)
	ST4471^*^ (1)	t17637^*^ (1)
CC25 (17)	ST25 (15)	t078 (7), t258 (3), t12584 (2), t280 (1), t7125 (1), t349 (1)
	ST4444^*^ (2)	t287 (2)
CC121 (5)	ST121 (4)	t2524 (3), t2091 (1)
	ST1301 (1)	t2524 (1)
CC72 (19)	ST72 (18)	t3092 (14), t1346 (4)
	ST4436^*^ (1)	t148 (1)
CC395 (2)	ST4434^*^ (1)	t16984^*^ (1)
	ST395 (1)	t17624^*^ (1)
CC97 (10)	ST97 (9)	t730 (3), t237 (2), t359 (2), t4682 (1), t267 (1)
	ST3257 (1)	t267 (1)
CC88 (42)	ST88 (41)	t1376 (13), t12444 (3), t16824 (2), t16751 (2), t5917 (2), t2592 (4), t6497 (2), t4016 (3), t2526 (1), t2421 (1), t3155 (1), t17633^*^ (3), t17640^*^ (2), t17626^*^ (1)
	ST4448 (1)	t4016 (1)
CC8 (7)	ST630 (5)	t377 (4), t4549 (1)
	ST8 (2)	t024 (2)
CC121 (13)	ST12 (12)	t213 (8), t4176 (1), t127 (1), t17630 (1), t17632 (1)
	ST1156 (1)	t213 (1)
Singletons (23)	ST2990 (5)	t091 (3), t1689 (1), t2883 (1)
	ST672 (2)	t003 (1), t3841 (1)
	ST2885 (2)	t13849 (2)
	ST1094 (1)	No-type
	ST504 (1)	t529 (1)
	ST45 (45)	t116 (3)
	ST1085 (1)	t208 (1)
	ST944 (1)	t616 (1)
	ST4475^*^ (1)	t5100 (1)
	ST4472^*^ (1)	t127 (1)
	ST4569^*^ (1)	t17707^*^ (1)
	ST4076^*^ (1)	t11641 (1)
	ST4075^*^ (1)	t16941^*^ (1)
	ST4469^*^ (1)	t17708^*^ (1)
	ST4468^*^ (1)	t045 (1)
	ST4071^*^ (1)	t16896^*^ (1)
	ST9 (30)	t899 (27), t100 (1), t127 (1), t4132 (1)
	ST692 (8)	t2247 (8)
	ST10 (1)	t528 (1)
	ST4063^*^ (2)	t189 (2)
	ST4456^*^ (1)	t13849 (1)
	ST4452^*^ (1)	t5502 (1)
	ST133(1)	t998 (1)

* *represents novel types*.

Using MLST there were 111 different sequence types (STs) were detected among the 868 *S. aureus* isolates analyzed, including 65 newly assigned STs which showed most of them was a single-locus variant (refer to Table 4). Based on eBURST analysis, the 111 STs were grouped into 20 clonal complexes (CCs) and 23 singletons (Table 4). Most CCs (CC1, CC7, CC15, CC5, CC188, CC398, CC6, and CC59) consisted of a central prevalent genotype associated with several much less-frequent single-locus variants (SLVs). The common allelic profiles (STs) was ST7 (151/868, 17.4%), followed by ST1 (124/868, 14.3%), ST5 (73/868, 8.4%), ST398 (59/868, 6.8%), ST188 (48/868, 5.53%), ST6 (42/868, 4.8%), ST59 (42/868, 4.8%), ST88 (41/868, 4.7%), ST15 (35/868, 4.0%), ST9 (30/868, 3.5%), ST72 (18/868, 2.1%), ST25 (15/868, 1.7%), ST943 (14/868, 1.6%), ST12 (12/868, 1.4%), and other STs (<1.0%). Most of the novel STs were represented by either two or one strains.

Combining the STs and *spa* types, there are 248 different ST-*spa* types in this research (Table 4). However, most isolates demonstrated high consistent between STs and *spa* types, such as ST1-t127 (93/868, 10.7%), ST7-t091 (92/868, 10.6%), ST5-t002 (42/868, 4.8%), ST398-t034 (40/868, 4.6%), ST188-t189 (38/868, 4.4%), ST59-t437 (30/868, 3.5%), ST6-t701 (29/868, 3.3%), and ST9-t899 (27/868, 3.1%), and other types. The different sampling regions or food types possibly affected which genotypes were found; for example, Sta353, Sta3802C1, and Sta4117A1 were isolated from different places and belonged to the same ST but were divided into different *spa* types (ST30-t021, ST30-t964, and ST30-t018). Some strains had the same *spa* type but differing STs (e.g., t127-ST1, t127-ST12, and t127-ST59).

**Figure 2:**
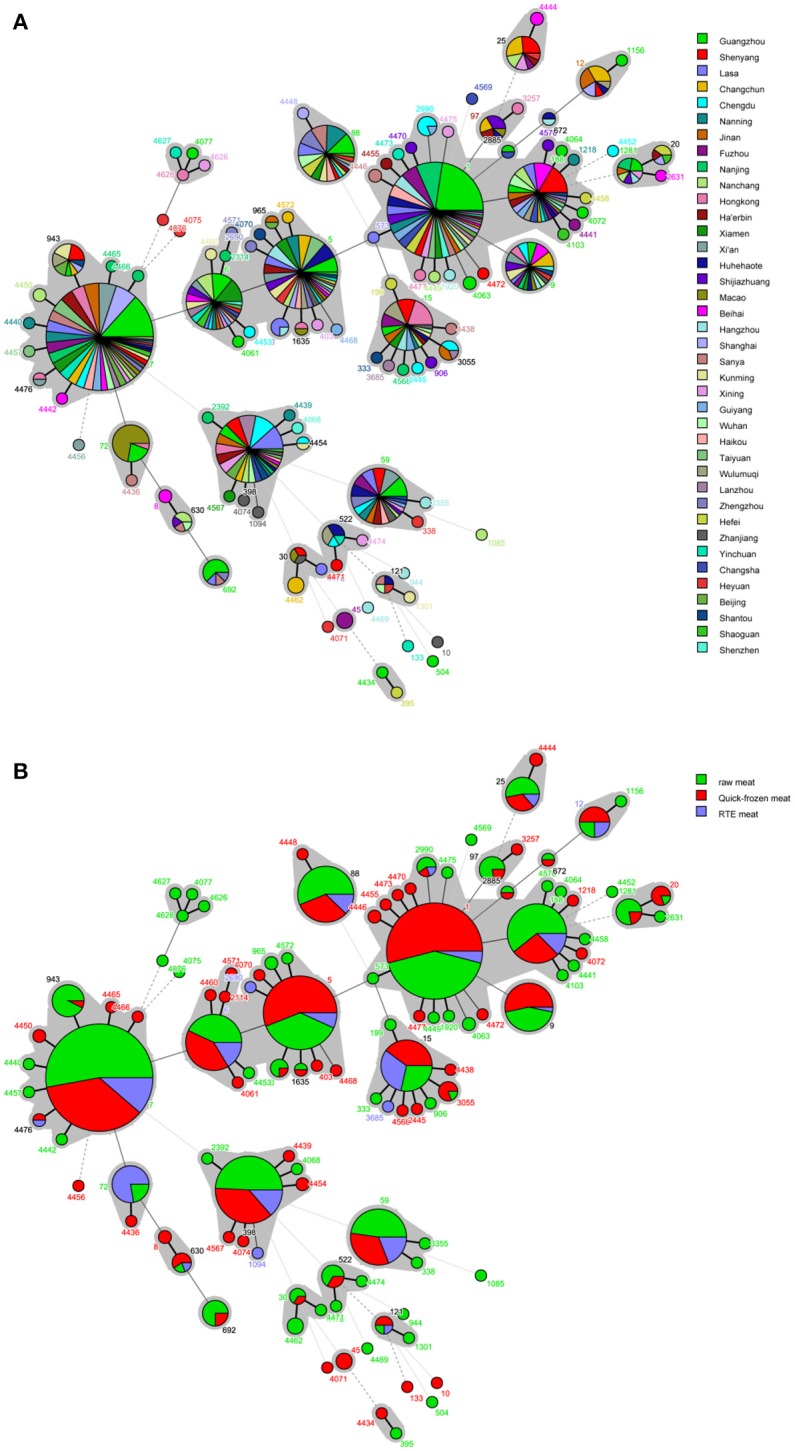
Minimum spanning tree of MLST data for 868 *S. aureus* isolates. Each circle represents one ST, the size of which is related to the number of strains within this ST, while the branch numbers account for the number of allele differences between connected STs. The colors in the circles in **(A)** represent the sampling locations, and the colors in the circles in **(B)** represent the food sources. The gray zones surrounding some STs indicate that these genotypes belong to the same clonal complex.

A phylogenetic tree based on the 7 concatenated MLST sequences shows the relatedness between the strains. The STs of *S. aureus* isolates were further analyzed based on the sampling sites and food sources (Figures 2A,B). All STs were fell into three different cladogram (designated as A, B, and C) which originate from CC7. Most novel were single-locus variant. Cladogram A contained CC6, CC5, CC1, CC188, ST97, ST20, ST25 and some relevant single-locus variant; Cladogram B included CC398, CC59, ST522, ST30 and some relevant single-locus variant; Cladogram C contained ST72, ST8, ST630, and ST692. It could be found that three different cladogram were evolved in directions, but distribute in different cities and different food sources, which showed high genetic diversity of *S. aureus* in China. The quick-frozen meat and raw meat were the major types in most of the CCs, whereas RTE meat was dominant for CC72. Interestingly, CC72 was only identified in *S. aureus* isolated from South China (Guangzhou, Macao, Hong Kong and Haikou).

## Discussion

Recent years, although many studies about the prevalence of *S. aureus* in retail food have been reported in China (Wang et al., 2012, 2013; Zhang et al., 2013; Chao et al., 2015), the investigation of qualitative and quantitative in national level are lacking. Therefore, a more comprehensively investigation for the incidence of *S. aureus* isolated from retail meat foods is needed. In this study, samples collected covered all provincial capitals of China, and the food types were also more diversification. Moreover, in conjunction with resistance properties and molecular types, it is better to understand the genetic background of these strains.

Overall, 35% of retail meat and meat products were positive for *S. aureus*. Over the 39 cities, *S. aureus* were both confirmed that ranging from 16.7% in Shenzhen to 48.8% in Beihai. This result suggests that the contamination of *S. aureus* was common in retail meat and meat product in China. This study also determined the contamination level of retail meat in China, showing that the MPN values of the majority of the positive samples between 0.3 and 110 MPN/g, resulting in a average value of 10.35 MPN/g. Among the 647 positive samples, 33 (5.1%) samples had levels beyond 100 MPN/g, whereas 319 (49.3%) had levels below 1 MPN/g. To our knowledge, a limit of 100 cfu/g had been proposed for *S. aureus* levels in raw meat and RTE meat in China (National Population and Family Planning Commission of the People's Republic of China, 2014). Thus, as a whole, the contamination levels of *S. aureus* in raw meat in China were not very excessive.

Compared with studies from other countries, the presence of *S. aureus* in retail meat was higher than the study of Ge et al. (2017) who reported that 27.9% of retail meat samples in the USA were positive for *S. aureus*, but lower than (Tang et al., 2017)'s research who reported that 68% of samples in Denmark were positive. In other researches, the prevalence of *S. aureus* in food ranged from 4 to 76.47% (Bakr et al., 2004; Kitai et al., 2005; van Loo et al., 2007; Buyukcangaz et al., 2013; Jackson et al., 2013; Bunnoeng et al., 2014; Dhup et al., 2015). Attribute to the sample sizes, sample types, and geographic locations of investigation, it is maybe the reason for these differences. Based on food categories, *S. aureus* was found in 51.0%. 43.35 and 12.2% of samples of raw meat, quick-frozen meat, and RTE meat, respectively. This result indicated that the processing can reduce the contamination of *S. aureus*. There was a previous study which summarized by Ou et al. (2017) in 2017, it was collected 39 studies of the prevalence rates of *S. aureus* contamination by different types of raw meat products across different periods, regions, and sampling locations and found that the prevalence of *S. aureus* contamination in chicken products was highest in Asian. In this study, it was similar to this research result, which showed the prevalence of *S. aureus* contamination in 67.9% of raw poultry and 60.9% of quick-frozen poultry. Besides, the prevalence of *S. aureus* in raw meat and quick-frozen meat was significantly higher than that in RTE meat (*p* < 0.001, χ^2^ test), however, the differences were not significant in the quantitative level (*p* > 0.05, χ^2^ test). At present, freezing is an excellent way to preserve food quality and to minimize the incidence of foodborne pathogens (Kumurya, 2013; Wu et al., 2015). Therefore, the cross contamination from environments (such as human, water or transportations) may be the major reason for the presence of *S. aureus*, which suggests that the Chinese food safety regulators should further improve their hygiene and supervision.

The resistance of *S. aureus* in this study was higher than that of other food pathogens such as *Salmonella, Listeria monocytogenes, Vibrio parahaemolyticus* in our previous study (Shi et al., 2015; Wu et al., 2015; Xie et al., 2016; Yang et al., 2016). However, many reports have detected antibiotic-resistant strains of *S. aureus* in various food products (Normanno et al., 2007; Basanisi et al., 2017; Rong et al., 2017), our data were similar to their results. In the current study, we detected only 11 isolates (1.26%) that were susceptible to all 24 tested antibiotics. The isolates of resistance or intermediary resistance to more than three or more antibiotics were reached 94.6% (821/868). Of which, 104 isolates (12.0%) were resistant to more than 10 antibiotics. In China, (Xing et al., 2014) revealed that 98.4% of *S. aureus* isolates in ready-to-eat food were resist to more than one antimicrobial agent and 58.6% of isolates resist to more than three antimicrobials, while (Hao et al., 2015) found that 35.9% of *S. aureus* isolated from quick-frozen dumpling were resistant to less than or equal to 3 kinds of drugs. In Wang et al. (2017) reported that 97.6% of *S. aureus* isolates from retail food showed resistance in one or more antimicrobial agents. The prevalence of resistance to clindamycin, gentamicin, penicillin, erythromycin, tetracycline, and ciprofloxacin is in agreement with our data. In addition, resistance to kanamycin, erythromycin, and tetracycline was common in our study. As important supplements for animal feed as growth promoters and for the treatment of human disease (Adzitey et al., 2013; Rasha et al., 2018), it maybe the major reason for more and more resistant strains have been commonly observed.

In this study, we also identified 62 MRSA isolates by the disk diffusion method and *mecA/C* detection. It showed 7.14% of MRSA in *S. aureus* isolates from retail meat. Most of the MRSA strains were resist to β-lactams and other antibiotics which showed 77.4% (48/62) were resistant to more than 10 antibiotics. This result as similar with the results of research by Wang et al. (2017) and Hao et al. (2015) in China. In fact, the prevalence of MRSA varies greatly by geographical location in retail meats. For example, MRSA was present in 1.9% of 3,520 retail meats in the USA (Ge et al., 2017), 0.5% (13/2,810) in Korea (Kim et al., 2015), 1.6% (5/318) in Spain, 13% in Denmark (19/145) (Tang et al., 2017), 11.9% in the Netherlands (264/2,217), and 24.8% in Canada (655/2,640) (Narvaez et al., 2016). Sample size, geographic area and collection period may responsible for the differences observed (Ge et al., 2017).

The genetic types of 868 *S. aureus* isolates acquired from raw meat and meat products in China were characterized by MLST and *spa* typing. Comparing the STs and *spa* types, it seems that *spa* typing had a superior distinguishability than MLST. In this study, more than one *spa* type was detected in the major of STs. This result is consistent with those of previous studies by Hata et al. (2010) and Song et al. (2015). In our study, despite the wide genetic diversity observed among the 868 *S. aureus* meat isolates, a great proportion of the population belonged to finite number of major clones: ST1, ST7, ST5, ST398, ST188, ST6, ST59, ST88, ST15, and ST9 (74.3%, 645/868) were the dominant STs in *S. aureus* from retail meat. In concordant with MLST types, t127, t091, t002, t189, t034, t701, t437, t899, t796, t084, t3092, t085, t164, and t1376 (63.7%, 553/868) were the dominant *spa* types. According to the *S. aureus* MLST database (https://pubmlst.org/bigsdbdb=pubmlst_saureus_isolates&page=profiles), there were 448 strains of ST1, 54 strains of ST7, 3,993 strains of ST5, 1,412 strains of ST398, 183 strains of ST188, 82 strains of ST6, 172 strains of ST59, 108 strains of ST88, 485 strains of ST15, and 50 strains of ST9 were demonstrating, the origins of them covered US, Canada, Poland, The Netherlands, Denmark, Australia, Bulgaria, Italy, Malaysia, Ivory Coast, France, Japan, Switzerland, and many other countries around world. Most of them isolated from the blood, pus, nasal swab, or skin swab. Beside, these types of *S. aureus* isolates have been relevant to a variety of clinical infections (Grundmann et al., 2010; Neocleous et al., 2010; Lozano et al., 2011; Valentindomelier et al., 2011; He et al., 2013; Suhaili et al., 2016). it indicates that these types of *S. aureus* strains have a theoretical pathogenic potential.

In Denmark, Tang et al. (2017) reported that the most frequent MRSA *spa* type and MSSA *spa* type were both CC398-t034. In USA, 25.9% of MRSA were identified as ST398-t034 (Hanson et al., 2011). In Germany, Feßler et al. (2011) also found 28 of 32 food related MRSA were CC398. In fact, CC398 strains are usually found in retail chicken, turkey, pork, and beef (Lozano et al., 2009; Huber et al., 2010; Argudín et al., 2011; Verhegghe et al., 2013), and have been perceived as a livestock-associated pathogens. Interestingly, we only found 7 isolates that were MRSA ST398, whereas MSSA ST398 was a common clone (52 isolates) in retail meat, and meat products from China in this study. Currently, infections caused by MSSA ST398 have been reported in humans and shown to cause infections more frequently than MRSA ST398 (Valentindomelier et al., 2011; Mediavilla et al., 2012; David et al., 2013; Li et al., 2015). In addition, the staphylococcal cassette chromosome mec (SCC*mec*) element types were diverse in MRSA ST398 (Song et al., 2015). In Price et al. (2012) collected 89 CC398 *S. aureus* isolates (including MSSA and MRSA) from animals and humans spanning 19 countries and four continents by whole-genome sequence typing and found that livestock-associated MRSA CC398 originated as MSSA ST398 in humans. The lineage appears to have undergone a rapid radiation in conjunction with the jump from humans to livestock, where it subsequently acquired tetracycline and methicillin resistance (Price et al., 2012). Therefore, when antibiotic selection in connection with food animal production, it maybe raise the potential chance of MSSA CC398 to acquire the SCC*mec* cassette (Song et al., 2015), however, further studies should be elucidate the possible rules for regarding MSSA ST398 as well.

In the present study, the majority types of MSSA was ST1-t127 (93/868, 10.7%) and ST7-t091 (92/868, 10.6%). In fact, ST7-t091 and ST1-t127 isolates have been reported as the fourth and sixth most prevalent clone, comprising both MSSA and MRSA strains, isolated from human invasive infections in 26 European countries (Grundmann et al., 2010). In Franco et al. (2011) demonstrated that t127/ST1 isolates can be assigned to two genetically different clusters (porcine and human) and hypothesized that ST1-t127 strains could represent another lineage of livestock-associated pathogens. On the other hand, CC59-t437 (41.9%, 26/62) was the predominant types in retail meat MRSA in China, followed by ST9-t899 (27.4%, 17/62). CC59-t437 is the predominant Asian community-associated MRSA (CA-MRSA) lineage, ranging from 35.8 to 76.7% with CA-MRSA in China (Yang et al., 2017). In Yang et al. (2017) were collected *S. aureus* strains in Beijing Children's hospital from respiratory tract, skin, and soft tissue, sterile sites in 104 children cases and found 61.7% of CA-MRSA was ST59-SCC*mec* IV-t437. Except China, ST59 was also reported in Vietnam, Japan, Australia, and other counties for CA-MRSA infection (Tang et al., 2007; Coombs et al., 2010; Higuchi et al., 2010). Therefore, further research will be proceed and found the reason of why ST59-t437 was the predominant types of MRSA isolates in retail meat in our study China. Besides, ST9-t899 was consider that the predominant *S. aureus* and MRSA genotype in pigs and related workers in Asia (Chuang and Huang, 2015). According to the previous studies, there were many studies have been reported the prevalence of this type of MRSA in Taiwan, Hong Kong, Malaysia, Thailand, and other countries from livestock (Neela et al., 2009; Graveland et al., 2011; Larsen et al., 2012; Lo et al., 2012). Thus, the majority types are different than those in studies in other counties. However, as well as livestock-associated lineages and clinical *S. aureus* clones, it revealed that these strains were widespread from retail meat and meat products in China. The pathogenic potential of these strains from retail meat food in China should not be ignored.

## Conclusions

In conclusion, we report a wide scale and systematic investigation of *S. aureus* from retail meat and meat products in China, it supplements the nationwide qualitative and quantitative data of the prevalence and levels of *S. aureus*. The contamination of *S. aureus* was common in retail meat in China, but the levels of *S. aureus* were not very excessive. The prevalence rate of *S. aureus* in raw meat and quick-frozen meat was significantly higher than that in RTE meat. Most *S. aureus* isolates exhibited resistance to a variety of antimicrobials. By molecular typing analysis showed that these isolates had a high genetic diversity. The majority of these types have been linked to human infections worldwide, indicating that *S. aureus* strains of these types in retail meat related *S. aureus* in China have at least a theoretical pathogenic potential. However, the frequently types in our study are different than those in studies in other counties. Thus, further studies may need to elucidate the potential origins of these strains in China.

## Author contributions

QW, JZ, SW, and TL conceived and designed the experiments. JH and FZ performed the experiments. SW, HW, and HZ analyzed the data. XY, LX, MC, YD, and SZ contributed reagents, materials, analysis tools. SW, JW, and QW contributed to the writing of the manuscript.

### Conflict of interest statement

The authors declare that the research was conducted in the absence of any commercial or financial relationships that could be construed as a potential conflict of interest.
